# Increased Frequency of Giant Miniature End-Plate Potentials at the Neuromuscular Junction in Diabetic Rats

**DOI:** 10.3390/biomedicines12010068

**Published:** 2023-12-27

**Authors:** Julián Elías Martínez-Sánchez, Yolitzy Cárdenas, Xóchitl Trujillo, Mónica Ríos-Silva, M. Irene Díaz-Reval, Jaime Alberto Bricio-Barrios, Jesús Muñiz, Julio Alcaraz-Siqueiros, Miguel Huerta

**Affiliations:** 1 Centro Universitario de Investigaciones Biomédicas, Universidad de Colima, Av. 25 de julio # 965, Col. Villas San Sebastián, Colima 28045, Colima, Mexico; julian_martinez@ucol.mx (J.E.M.-S.); rosio@ucol.mx (X.T.);; 2 Centro Universitario de Investigaciones Biomédicas, Universidad de Colima—CONAHCyT, Av. 25 de Julio 965, Col. Villas San Sebastián, Colima 28045, Colima, Mexico; 3 Facultad de Medicina, Universidad de Colima, Av. Universidad #333, Col. Las Víboras, Colima 28040, Colima, Mexico; jbricio@ucol.mx; 4 Facultad de Ciencias Biológicas y Agropecuarias, Universidad de Colima, Km 40 Autopista Colima-Manzanillo, Crucero de Tecomán, Tecomán 28930, Colima, Mexico

**Keywords:** diabetes mellitus, Wistar rat, MEPPs, GMEPPs, neuromuscular junction

## Abstract

There is a need for research addressing the functional characteristics of the motor end-plate in diabetes to identify mechanisms contributing to neuromuscular dysfunction. Here, we investigated the effect of diabetes on spontaneous acetylcholine release in the rat neuromuscular junction. We studied two randomized groups of male Wistar rats (n = 7 per group, 350 ± 50 g, 12–16 weeks of age): one with streptozotocin-induced experimental diabetes, and a healthy control group without diabetes. After 8 weeks of monitoring after diabetes induction, rats in both groups were anesthetized with pentobarbital. Then, the diaphragm muscle was dissected for electrophysiological recordings of miniature end-plate potentials (MEPPs) using a single electrode located at the region of the muscle end-plate. All experiments were conducted at environmental temperature (20–22 °C) in rat Ringer solution with constant bubbling carbogen (95% O_2_, 5% CO_2_). Compared to healthy controls, in the diaphragm neuromuscular end-plate derived from diabetic rats, the MEPPs were higher in amplitude and frequency, and the proportion of giant MEPPs was elevated (7.09% vs. 1.4% in controls). Our results showed that diabetes affected the acetylcholine MEPP pattern and increased the number of giant potentials compared to healthy controls.

## 1. Introduction

Type 2 diabetes mellitus (T2DM) is a chronic degenerative disease characterized by metabolic failure caused by the combination of two factors: a reduction of insulin, and tissues developing a decreased or null sensitivity to insulin. These factors lead to dysregulation of glucose homeostasis [1]. Insulin is a peptide hormone produced and secreted by β-pancreatic cells when glucose increases in blood. Insulin acts on metabolic tissues—including the liver, skeletal muscle, and adipose tissue—to obtain glucose from glycogen or lipids. This leads to increased glucose levels in the blood, and glucose crosses the blood–brain barrier, exerting effects on memory and learning [2].

T2DM results in constant hyperglycemia, which can lead to several types of organ and tissue failures, resulting in various health complications; incapacitating effects; microvascular complications, such as retinopathy, nephropathy, and neuropathy; and macrovascular complications with a higher probability of developing a cardiovascular disease [1,2,3]. This disease can cause dysfunction of the renal tissue, retina, cardiovascular system, liver, and neurons [2,3,4]. Once diagnosed, diabetes is rarely reversible. Insulin also crosses the blood–brain barrier, regulating key central nervous system functions in persons with diabetes [4].

Diabetes mellitus is a global health epidemic [5], and T2DM has become the ninth leading cause of death. In 2017, this disease affected about 462 million people, which is equivalent to about 6.28% of the world’s population. The T2DM prevalence rate is 6059 cases per 100,000 inhabitants. The incidence is similar between sexes and is higher among individuals over 50 years of age (15% in the age group of 50–69 years, and 22% among those over 70 years old). Over one million deaths per year could be caused by diabetes alone. It has been predicted that by 2030, the global prevalence of T2DM will increase to 7079 cases per 100,000 individuals, with a continuous increase in each region of the world [1,6]. The prevalence of T2DM in the young population is concerning, as this disease is more aggressive in young people than among adults [7]. In Mexico, T2DM affects 14.4% of the adult population over 20 years old, and over 30% of persons over 50 years old [8].

Among the comorbidities associated with DM, diabetic polyneuropathy (PND) is a common condition, characterized by sensory and motor dysfunction. The motor deficits in patients with diabetic neuropathy have been of interest and extensively studied; however, the thought is that diabetic neuropathy has lesser effects on the motor system than on the sensory system [4]. Therefore, research on PND often focuses on the sensory components, while underestimating the effects on the motor and neuromuscular systems [9]. Notably, the combination of sensory deficits with motor system changes may contribute to decreased functional ability (capacity), impaired mobility, gait disturbances, and increased risk of falls [10,11,12]. Thus, there is a need for experimental studies that address the morphofunctional characteristics of the neuromuscular junction (NMJ) and focus on intrinsic skeletal muscle problems [13] for the identification of other mechanisms that contribute to DM-related motor dysfunction [14].

The neuromuscular junction (NMJ) is a specialized zone called a synapse that connects the motoneuron ending with one or more muscle fibers [15]. In our present study, we focused on the skeletal muscle NMJ, where an impulse transmitted by acetylcholine to the muscle conducts a depolarization that generates a new electrical impulse in the muscle fiber [13,14,15]. Diverse pathologies lead to the development of lesions in skeletal muscle, including muscle dystrophia and sarcopenia. The effects of these pathologies are not limited to intrinsic processes of the muscle but can also influence denervation and changes in the fragmentation of the NMJ, which modifies the transmission of neuromuscular information [15]. Changes in the NMJ can impact motoneuron functionality. The disease myasthenia gravis is an example of this failure, which leads to increased muscle debility and fatigue of skeletal muscle [15].

The original evidence of quantum release was obtained from studies of the NMJs from frogs [16]. At the NMJ, it is possible to record a series of depolarizations in the absence of stimulation. These depolarizations—which are called miniature end-plate potentials (MEPPs)—are similar in shape to the neuromuscular end-plate potential but with an amplitude 50 times smaller. MEPPs were first discovered in frog NMJs by Fatt and Katz [16,17] and were subsequently also described in rat NMJs by Liley [18]. Since then, MEPPs have attracted research interest because only nicotinic ACh receptors take part in MEPP generation, making them valuable for studies of ACh channel function [17,18,19]. In addition to controlling neuromuscular synaptic transmission, nicotinic receptors modulate the activity of several neuronal circuits [20].

In this present study, we aimed to investigate how diabetes mellitus (DM) affected spontaneous ACh release in NMJs obtained from the muscles of rats. Our findings will help to elucidate the alterations associated with neuromuscular dysfunction in DM, and aid in the determination of a possible underlying mechanism.

## 2. Materials and Methods

### 2.1. Animals and Diabetes Induction

The experiments were conducted on 14 male adult Wistar rats (12–16 weeks old, weighing 350 ± 50 g) provided by the animal facility of the University Center for Biomedical Research from the University of Colima. These rats were randomly divided into two groups: diabetic and control (n = 7 each). The sample size was calculated using the Arifin formula, with consideration of the “3Rs” (Replacement, Reduction, Refinement) animal use alternatives [21,22]. The rats were group-housed in 47 × 37 × 19 cm polypropylene cages, with the floor covered with sterilized wood shavings. The chip change was carried out daily, and in the case of diabetic rats, the change was carried out twice a day. The rats were kept in a controlled environment with day cycles of 12 h:12 h of light/dark, at a temperature of 23 ± 2 °C, and with a relative humidity of 40%. The animals were fed ad libitum with a standard diet, Purina^®^ brand nutricubes, and purified water. Food was provided every day, and water was changed daily by washing and refilling the jars to avoid the accumulation of fungi in the drinker.

All animals were kept following the Institutional Animal Care and Use Committee and in agreement with the Official Mexican Standard NOM-062-ZOO-1999 guidelines for the care and handling of experimental animals in the animal facility of the University Center for Biomedical Research at the University of Colima.

### 2.2. Induction to Diabetes

For the induction of diabetes, the rats were fasted overnight for 15 h. The induction of experimental diabetes was conducted with streptozotocin (STZ) (Sigma-Aldrich Co., St. Louis, MO, USA), which is an antibiotic but has direct effects on pancreatic β cells, causing their destruction. STZ has been widely used as a model of experimental diabetes in animals [23].

In the diabetic group, diabetes was induced by a single intraperitoneal injection of streptozotocin (STZ) (45 mg/kg, freshly prepared in 0.1 M citrate buffer, pH 4.5) [24,25]. Seven days after induction, blood glucose was measured at the tail vein using a glucometer (Accu-Chek; Roche Diagnostics, Indianapolis, IN, USA). Rats with a blood glucose higher than 200 mg/dL after an overnight fast were considered diabetic [23,24]. After diabetes confirmation, both the healthy control group and the diabetes group were kept for 8 weeks with food and water ad libitum.

### 2.3. Diaphragm Dissection

From the two groups of male Wistar rats, we obtained isolated phrenic nerve preparations. After 8 weeks of diabetes induction, the animals were anesthetized with an i.p. injection of pentobarbital sodium (60 mg/kg) [26] and euthanized by cervical dislocation [27]. Immediately after euthanization, the diaphragm was removed and rinsed with the oxygenated (95% O_2_–5% CO_2_) Ringer solution (136.8 mM NaCl, 5 mM KCl, 1 mM MgCl_2_, 15 mM NaHCO_3_, 1 mM Na_2_HPO_4_, 11 mM D-glucose, and 2.0 mM CaCl_2_, pH 7.4) [27,28,29].

After removing the diaphragm muscle from the rat, we transferred the diaphragm to the dissection plate. The dissection plate is a 120 mm petri dish (USA Scientific, Ocala, FL, USA) filled with Sylgard (Dow Corning, Midland, MI, USA) to a height of 5 mm. An oxygen bubbler was placed on top of the Sylgard to oxygenate the Ringer solution during the diaphragm dissection. The oxygen bubbler was secured to the dissection plate with surgical tape. The diaphragm muscle was pinned using 0.2 mm diameter stainless steel pins (Carolina Biological Supply, Burlington, NC, USA) onto a transparent Sylgard block. The diaphragm muscle was covered with the oxygenated Ringer solution throughout the dissection procedure [28]. While viewing through a stereomicroscope (Nikon, Melville, NY, USA), we carefully removed fat and connective tissues. Each diaphragm was isolated and placed in a Sylgard-coated Petri dish having Ringer solution with constant bubbling of a mixture of 95% O_2_ and 5% CO_2_, at room temperature (22–23 °C) [30]. We transferred the preparations to the electrophysiology setup to perform intracellular recordings of spontaneous MEPPs.

### 2.4. Electrophysiological Recording

MEPPs were recorded in a conventional way [16] with single Ag-AgCl filament intracellular electrodes (A-M Systems, Inc., Sequim, WA, USA), filled with KCl (3 M) and 10–20 MΩ resistance [30,31], inserted in the motor end-plate. The muscle fibers used for recording had a resting membrane potential (RMP) of more than −65 mV, and values were continuously monitored during the experiment using the pCLAMP 10 Clampex software version 9.2 (Axon Instruments, Foster City, CA, USA).

The muscle fiber was selected before recording, which had a first membrane potential. Once MEPPs were detected, a threshold for MEPP detection was set up at an amplitude within the range of 0.25 mV to <1 mV, while the threshold for giant MEEP (GMEPP) detection was set as >1 mV. Detection of MEPPs was performed through event protocol detection. To avoid contamination of the recording signal by electrical noise, we considered for the measurements only events with a duration of >2 ms [27]. For each end-plate synaptic potential, the recording period was 2 min. For data analysis, we used the pClamp 10.5 software (Axon Instruments, Foster City, CA, USA).

### 2.5. Statistical Analysis

The data expression is the mean ± SEM across experiments. To find statistical differences in the results, comparisons were made using Student’s *t*-test for independent samples. A *p*-value of <0.05 was considered significant. The data were analyzed using Rstudio and GraphPad Prism v.9 software.

## 3. Results

### 3.1. Glucose and Weight of Wistar Rats

After diabetes confirmation (fasting blood glucose ≥200 mg/dL), weight and glucose were evaluated. The initial measurements were taken at the beginning of the 8 weeks, and the final measurements at the end of the 8 weeks. Table 1 presents the body weights and serum glucose levels of the rats at the beginning and end of the study. Body weight in the diabetic rats was higher than in healthy rats at the beginning of the study and had significantly decreased at the end of the study, which was expected due to diabetes (351 ± 19 g vs. 306 ± 26 g; *p* < 0.001) (Table 1). Fasting glucose was measured after 8 h overnight fasting. In healthy rats, fasting glucose was 79 ± 3 mg/dL at the beginning of the study, and then 72 ± 3 mg/dL at the end of the 8-week observation period. In the diabetic rats, the initial and the final glucose levels were 372 ± 15 mg/dL and 268 ± 17 mg/dL, respectively.

### 3.2. GMEPP Frequency Increases in Diabetic Wistar Rats

We obtained 73 useful recordings from each study group. Recordings were obtained from a total of 38 muscle fibers (19 from each group) isolated from the rats’ diaphragms (n = 14 total, n = 7 per group). All experiments were carried out at room temperature in the presence of the constant bubbling of a mixture of carbogen solution. The standard time for recording was 60 min.

**Figure 1:**
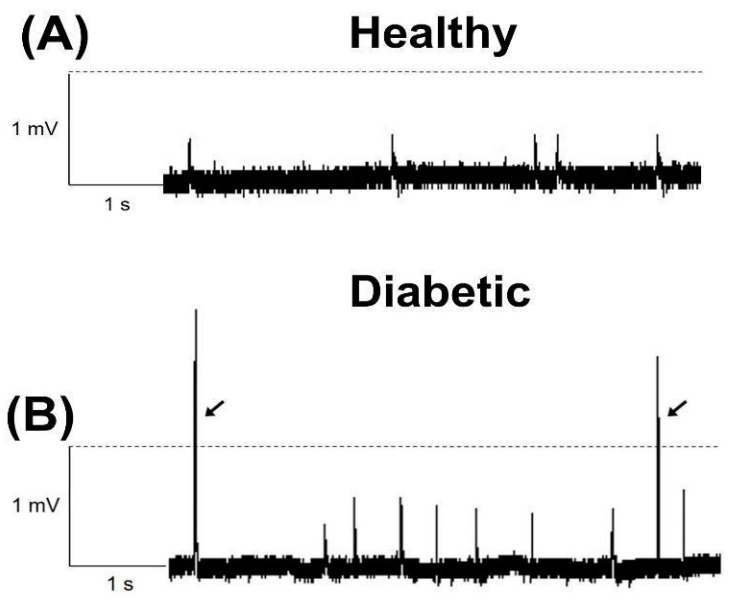
Representative miniature end-plate potential (MEPP) recordings from the neuromuscular junction (NMJ) of healthy (**A**) and diabetic (**B**) rats. Note the giant MEPPs (GMEPPs) (≥1 mV) from diaphragm NMJ derived from diabetic rats.

MEEP recording was performed at the end of the 8 weeks after diabetes confirmation. Figure 1 shows representative recordings of potentials from diaphragms of the healthy rats (Figure 1A) and diabetic rats (Figure 1B). These results showed how diabetes affected the frequency of recorded MEPPs, compared to recordings from NMJs derived from healthy rats. In the NMJ fibers from diabetic rats, we frequently observed spontaneous GMEPPs with amplitudes higher than 1 mV (Figure 1B).

**Figure 2:**
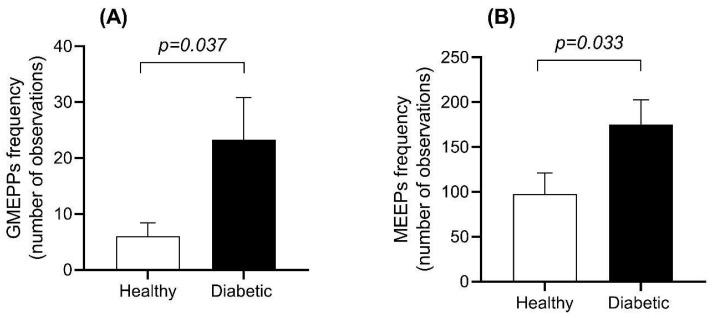
(**A**) Recording of giant miniature end-plate potentials (GMEPPs ≥1 mV) from the neuromuscular junction of the diaphragm from healthy and diabetic rats. (**B**) Recording of miniature end-plate potentials (MEPPs; <1 mV) from the neuromuscular junction of the diaphragm from healthy and diabetic rats. An increased frequency of GMEPPs is observed in the diaphragm of rats with diabetes. Data were from events obtained from 31 fibers per group (n = 7 rats per group). Graphs show the mean ± standard error. Comparisons were made using Student’s *t*-test for independent samples.

The frequencies of the MEPP and GMEPP potentials were independently analyzed. The numbers of observed GMEPPs and MEPPs were higher in the diabetic rats compared to healthy control rats (Figure 2A,B).

### 3.3. Diabetes Changes Electrophysiological Parameters of the NMJ

Diabetic rats exhibited a diminished resting membrane potential (RMP) of the diaphragm muscle fibers compared to healthy control rats (−75 ± 0.13 mV vs. −69 ± 0.24 mV; *p* < 0.001, Welch’s *t* test; Table 2). The time to peak in the GMEPPs recorded from muscle fibers of the diaphragm did not significantly differ between diabetic rats versus healthy control rats: 3.21 ± 0.06 ms (n = 38) vs. 3.14 ± 0.02 ms (n = 572); *p* > 0.05, Welch’s *t* test (Table 2). In contrast, in the MEPPs, we observed a significantly increased time to peak in those recorded from diabetic rats compared to healthy control rats: 2.7 ± 0.01 ms (n = 2709) vs. 2.8 ± 0.07 ms (n = 8065); *p* < 0.001, Welch’s *t* test (Table 2).

**Figure 3:**
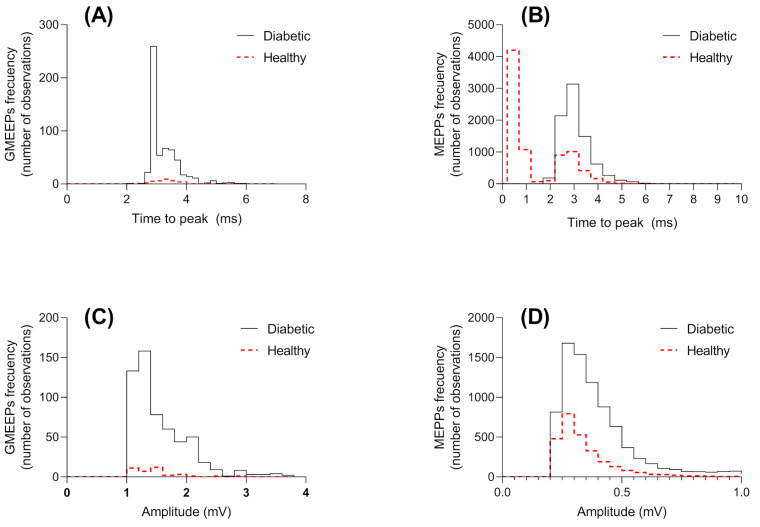
Histograms of miniature end-plate potentials (MEPPs) and giant MEPPs (GMEPPs) recorded from diaphragm muscle fibers at neuromuscular junctions (NMJs) derived from healthy rats (continued lines) and diabetic rats (punctuated lines). (**A**) Time to peak of GMEPPs. (**B**) Time to peak of MEPPs. (**C**) GMEPP amplitudes. (**D**) MEPP amplitudes. Data were from events obtained from 31 fibers per group (n = 7 rats per group).

Analysis of the recorded GMEPPs revealed that the frequencies differed between healthy rats (1.4%) and diabetic rats (7.09%). Figure 3 shows the distribution graphs of the times to peak and amplitudes for GMEPPs and MEPPs in healthy and diabetic rats.

## 4. Discussion

It is widely known that diabetes mellitus is related to motor deficits, as observed in diabetic patients with neuropathy due to axonal damage of motor neurons [32]. Several studies conducted in rodents and humans with diabetes have explored the changes in axonal morphology and in the ultrastructure of the NMJ, as well as motor function [10,33,34]. However, few studies have focused on alterations of neuromuscular transmission in this condition. Therefore, in our present study, we explored the effects of diabetes on Ach spontaneous release in the NMJ of mice with experimentally induced diabetes.

It has been reported that diabetic neuropathy has a relationship with axonal changes manifested in a decrease of peripheral nerve conduction velocity, which affects the F-wave presence and lengthens its latency, which explains, in part the demyelination and axonal damage of the phrenic nerve in diabetic rats [29,35]. Furthermore, it is possible that the affectation of NMJ is related to the time of diabetes evolution and hyperglycemia [36]. However, to date, there exist scarce studies that examine the functional and morphological changes in NMJ [35]. One of the reports suggests that diabetes could affect the phrenic nerve, causing respiratory complications [37]. In addition, a significative reduction in the transversal surface of the phrenic nerve has been observed in diabetic animal models [35,38], and a diaphragm with lipid accumulation in the red fibers associated with partial atrophy of the white fibers but no evaluation of the diaphragmatic function was performed before animal euthanasia [38]. The physiological implication in the animal is not well understood, which is a focus line for future studies.

Diabetes is known to affect postural muscles (mostly slow twitch) and to produce a reduction in respiratory muscle strength and diaphragm atrophy in animals and humans [39,40,41]. Here, we explored the release of ACh transmitter by recording the amplitude and frequency of MEPPs in diaphragm muscles isolated from rats with streptozotocin-induced diabetes. We found that compared to healthy control rats, diabetic rats exhibited significant increases in both MEPP frequency and amplitude, which are two of the first signs of neuronal injury [27,42].

Our results showed that the frequency of giant miniature potentials (GMEPPs ≥ 1 mV) was increased in the diaphragm muscles of diabetic rats compared to healthy control rats. Under normal conditions, these GMEPPs constitute between 1 and 2% of the total miniature potentials per unit of time [43] and appear much more frequently during degenerative and/or remodeling processes. Similarly, an increased frequency of GMEPPs has been reported during aging [27]. This phenomenon could be explained by a temporary summation mechanism or by constitutive vesicular secretion processes that involve mechanisms related to PKC in the regulation of vesicular recycling [44]. It has also been reported that the protein kinase CsNK2/CK2 plays an important role in maintaining a high density of acetylcholine receptors (AChRs) [45]. However, the exact mechanism that gives rise to these potentials in T2DM is currently unknown. Cyclic AMP activates kinase proteins (AMPK) and acts as an energetic sensor, with aberrant expression in tissues that suffer from several diseases, including diabetes mellitus. AMPK signaling could improve diabetes complications, such as neuropathy [46,47]. To our knowledge, this is the first time that GMEPPs have been reported in animal models of diabetes.

The amplitude of a potential is the difference, measured in mV, between the resting membrane potential and the higher value reached during depolarization. This value is associated with postsynaptic activity due to the activation of nicotinic channels in the muscle membrane, caused by the coupling of acetylcholine molecules to AChRs [48]. Our present results demonstrated that the distributions of amplitudes of GMEPPs and MEPPs differed between healthy and diabetic rats (Figure 3C,D). Doherty previously reported that hypertonic sucrose media was associated with increases in the amplitudes and frequencies of MEPPs, indicating that hypertonicity has a direct effect on this action. It has also been reported that amplitude changes can occur due to hypertonicity, which induces the generation of big Ach vesicles that generate changes in the nerve endings, increasing the ACh concentration or acting on cofactors that influence the vesicles [49].

The time to the peak of the potential is the difference between the time at which the amplitude at the peak is reached and the time at the beginning of membrane depolarization. It has been reported that the time to reach the higher amplitude is increased under conditions of NMJ injury [50]. Table 2 shows the mean time to peak among the MEPPs in the groups of healthy and diabetic rats (2.7 ms and 2.8 ms, respectively), showing a significant difference even though the values are very similar. However, Figure 3B shows that the distribution of the time to peak among healthy rats is bimodal, with the time to peak most frequently being less than 1 ms. This indicates that the time to peak for the MEPPs among diabetic rats was about three times higher than the time to peak for the MEPPs among healthy rats. This suggests that the time to depolarization of the NMJ is higher under pathological conditions. This phenomenon has also been observed upon knock-out of the NAMPT molecule [51] and of the desmin protein, which is known for thr enrichment of the synaptic membrane in the NMJ [52].

It seems that in the increased neurotransmission and in the increased frequency, both GMEPPs are necessary to preserve effective neuromuscular transmission in diabetic animals. There is a need for further research aiming to elucidate the mechanisms that underlie these adaptations and their progress throughout the time course of this disease. Another characteristic of MEPPs recorded from tissues damaged by diabetes is their increased frequency in NMJs and neurons. The large quanta produced by hypertonic treatment shares some common characteristics with the giant MEPPs reviewed by Thesleff [53]. Another study of mouse NMJs also used streptozotocin (STZ) to induce diabetes, but the dose used to produce diabetes was 200 mg/mL STZ and the animals had 7 months of age—age being a determinant factor for changes in the frequency of the MEPPs and the presence of GMEPPs—and that study did not report the increment in the number of GMEPPs [54].

Finally, these results stand for the exploration of the dynamic spontaneous neurotransmitter release measured through MEPPs in the absence of a stimulus, them being interesting for the knowledge of this phenomena in pathological conditions such as diabetes. However, more research needs to be elucidated about those changes at functional, morphological, and molecular levels. In the next step, these should be considered under the focus of stimulation and acetylcholine release, with the possibility of evaluating the integrity of NMJ in the diabetic condition.

## 5. Conclusions

Our results showed that diabetes affected the acetylcholine release pattern (MEPPs) and increased the constitutive Ach release (GMEPPs) compared to healthy controls.

## Figures and Tables

**Table 1:** Body weight and serum glucose levels in healthy and diabetic rats.

	Initial	Final
	Body weight (g)	
Healthy	326 ± 13	375 ± 31 *
Diabetes	351 ± 19	306 ± 26 *
	Fasting glucose (mg/dL)	
Healthy	79 ± 3	72 ± 3
Diabetes	372 ± 15	268 ± 17 ***

Data are presented as mean and mean standard error. * *p* < 0.05, *** *p* < 0.001 Student’s *t*-test for paired sample, n = 7.

**Table 2:** Electrophysiological characteristics of miniature end-plate potential (MEPPs) and giant MEPPs (GMEPPs) in the healthy and diabetic conditions.

	Healthy	Diabetic
Resting membrane potential (mV)	−75 ± 0.13	−69 ± 0.24 **
Time to peak (ms) GMEEPs	3.10 ± 0.06 (n = 38)	3.13 ± 0.02 (n = 572)
Time to peak (ms) MEEPs	2.7 ± 0.01 (n = 2709)	2.8 ± 0.07 *** (n = 8065)
Amplitude (mV) GMEEPs	1.37 ± 0.06 (n = 38)	1.47 ± 0.02 (n = 572)
Amplitude (mV) MEEPs	0.31 ± 0.002(n = 2709)	0.36 ± 0.001 *** (n = 8065)

Data are presented as mean and mean standard error. ** *p* < 0.01, *** *p* < 0.001 Welch’s *t*-test for independent sample, n = number of events in 31 fibers from seven rats per group.

## Data Availability

Data are available from the corresponding author upon request.
